# Data on the Distribution and Ecology of *Pistia stratiotes* L. (Araceae) in Hungary

**DOI:** 10.1002/ece3.73488

**Published:** 2026-04-30

**Authors:** Szabolcs Kis, Attila Molnár V.

**Affiliations:** ^1^ HUN‐REN–UD Conservation Biology Research Group, Department of Botany, Faculty of Science University of Debrecen Debrecen Hungary; ^2^ Department of Pharmacognosy, Faculty of Pharmacy University of Debrecen Debrecen Hungary; ^3^ Juhász‐Nagy Pál Doctoral School University of Debrecen Debrecen Hungary

**Keywords:** aquatic plant invasion, canal networks, clonal reproduction, hydrological dispersal, size‐dependent flowering, thermally influenced waters

## Abstract

Biological invasions by free‐floating macrophytes are emerging as a major threat to lowland freshwater systems in Central Europe, particularly where thermally influenced canal networks create novel habitat conditions. Here, we provide the first detailed account of the distribution, habitat characteristics, and reproductive performance of *Pistia stratiotes* along the Élővíz Canal system near Békés and Békéscsaba (SE Hungary), situated close to the northern margin of the species' European range. In September 2022, we surveyed 71 sampling points along the canal and its tributaries; *P. stratiotes* was recorded at 35 sites (49.3%; Table 1). Sampling locations where *P. stratiotes* was not detected are provided in Table S1. Occupied localities were characterised by chemically relatively homogeneous, strongly alkaline waters (pH 9.28–9.69) with low to moderate conductivity and slow flow (0.05–0.32 m s^−1^). Drift counts revealed that higher current velocities substantially enhanced downstream transport of rosettes, indicating that flow‐mediated dispersal is a key mechanism of spread within the canal network. Of 126 individuals examined, 12.7% were flowering. Reproductive plants had markedly larger leaves and produced significantly more vegetative ramets than non‐flowering individuals, suggesting a size‐dependent threshold for sexual reproduction coupled with enhanced clonal propagation under favourable conditions. Our findings demonstrate that partially thermally influenced lowland canals can support persistent *P. stratiotes* populations under temperate climatic conditions and may potentially act as invasion hubs, although this role was not explicitly tested in the present study. These systems, therefore, warrant priority for monitoring and early management interventions.

## Graphical abstract

Free‐floating macrophyte invasions pose an increasing threat to lowland freshwater systems in Central Europe, particularly in thermally influenced canal networks. This study provides the first detailed assessment of the distribution, habitat conditions, dispersal, and reproduction of *Pistia stratiotes* along the Élővíz Canal in southeastern Hungary, where the species was recorded at nearly half of the surveyed sites and showed flow‐mediated dispersal and size‐dependent reproduction. The results indicate that thermally altered canals can sustain persistent populations under temperate climates and may function as invasion hubs, highlighting the need for targeted monitoring and management.

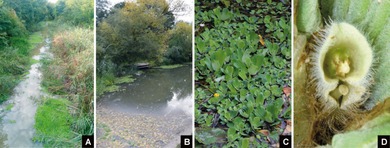

## 1 Introduction

Biological invasions by alien plant species represent one of the major drivers of biodiversity loss and ecosystem alteration worldwide (Pyšek et al. 2012; Simberloff et al. 2013; Kalusová et al. 2024), with freshwater habitats being particularly vulnerable because of their high connectivity and frequent anthropogenic disturbance (Chapman et al. 2020; Haubrock et al. 2023). Aquatic ecosystems are disproportionately affected by biological invasions (Havel et al. 2015), as water‐mediated dispersal facilitates rapid spread, while altered hydrological regimes, eutrophication, and climate change increasingly favour non‐native macrophytes over native vegetation (Hofstra et al. 2020).

Among freshwater environments, lowland canal networks function as effective invasion corridors, linking thermally and chemically distinct water bodies and providing stable habitats for free‐floating aquatic plants (Dorotovičová 2013; Anđelković et al. 2022). The ongoing warming trend associated with global climate change further amplifies invasion risks, particularly by species originating from (sub‐)tropical climatic zones, which are progressively overcoming former thermal constraints in temperate regions. This process is especially pronounced in thermally abnormal waters, that is, water bodies that are warmer than expected under local climatic conditions because of natural or anthropogenic influences, including thermal springs, effluents, and channels partly fed by thermal waters, which may serve as stepping stones for acclimatisation, long‐term persistence, and, potentially, evolutionary adaptation of warm‐adapted alien species (Hussner 2014; Hussner et al. 2014; Šajna et al. 2007, 2023). In this context, it is important to distinguish between short‐term physiological acclimation, phenotypic plasticity, and longer‐term evolutionary adaptation, which cannot be assessed on the basis of the present data. Recent records from Hungary (Lukács et al. 2016; E‐Vojtkó et al. 2017) and neighbouring Central European countries (Hrivnák et al. 2019; Šajna et al. 2023) highlight the growing importance of thermally influenced waters in the establishment of alien aquatic plants. The recent detection of *Eichhornia crassipes* (Mart.) Solms (Dudáš et al. 2025) and *Cabomba caroliniana* A. Gray (Takács et al. 2025) in partially thermally influenced canals in Békés County demonstrates that even species with high thermal demands can temporarily or persistently colonise artificial water bodies under favourable conditions, while also highlighting the importance of such habitats in enabling the spread of neotropical macrophytes within the Carpathian Basin. Within this context, water lettuce, *Pistia stratiotes* L. (Araceae), represents one of the most ecologically impactful free‐floating aquatic invaders worldwide (Xiong et al. 2023; Shen et al. 2025). It is a free‐floating perennial aquatic macrophyte whose introduced populations are now found in North America, Europe, Asia, Africa, and Oceania (Madeira et al. 2022), although it is most likely native to South America (Hussner et al. 2014). The species forms dense surface mats, suppresses native macrophytes, alters light regimes, oxygen dynamics and nutrient cycling, and significantly affects aquatic biota (Jaklič et al. 2020). Its invasion success is attributed to high phenotypic plasticity, rapid vegetative reproduction, buoyant dispersal units, and a broad tolerance of environmental conditions (Den Hollander et al. 1999; Galal et al. 2019).

In Central Europe, occasional occurrences of *Pistia stratiotes* as a casual alien (i.e., a species that may reproduce occasionally outside cultivation but does not form self‐sustaining populations; Richardson et al. 2000) have also been reported from non‐thermal water bodies, where populations usually remain ephemeral and fail to overwinter (Kaplan et al. 2016; Kis et al. 2026). Long‐term survival of *P. stratiotes* has been documented primarily in thermally influenced waters, such as thermal streams and warm‐water effluents (Šajna et al. 2007, 2023).

In 2022, we detected significant and spatially extensive populations of *Pistia stratiotes* in the Élővíz Canal system near Gyula and Békéscsaba, a partly thermal‐influenced canal network in Békés County. Although no documented record of this occurrence exists in the botanical literature, the website of the Federation of Angling Clubs of the Körös Region (see web source [1]) (along with the photographs published there) indicates that by 2013 the species already occurred in such quantities in the Élővíz Canal in Békéscsaba that a local environmental NGO (Zöld Csütörtök Környezetvédelmi Kör) organised a community action for its removal. According to local residents, despite repeated removal and thinning efforts by volunteers, the species has been observed every year since.

The apparent persistence of populations over multiple years at the climatic margin of the species' range raises important questions regarding habitat suitability and ecological tolerance under temperate conditions. Although such environments may favour the persistence of warm‐adapted species, assessing whether they promote evolutionary responses, such as increased cold tolerance, will require targeted physiological and genetic studies.

The aim of the present study was, therefore, to provide detailed data on the distribution, habitat characteristics, and ecological performance of *Pistia stratiotes* along the Élővíz Canal. The study is primarily descriptive in nature and aims to provide baseline ecological data on the occurrence, habitat characteristics, and reproductive traits of the species in a thermally influenced canal system. Specifically, we (i) mapped the spatial occurrence and extent of populations, (ii) characterised key abiotic parameters of occupied habitats, (iii) assessed hydrological drivers of local dispersal, and (iv) analysed size‐dependent flowering and vegetative reproduction to better understand the establishment potential of this invasive species at the northern edge of its European distribution.

## 2 Materials and Methods

### 2.1 Field Work

**FIGURE 1:**
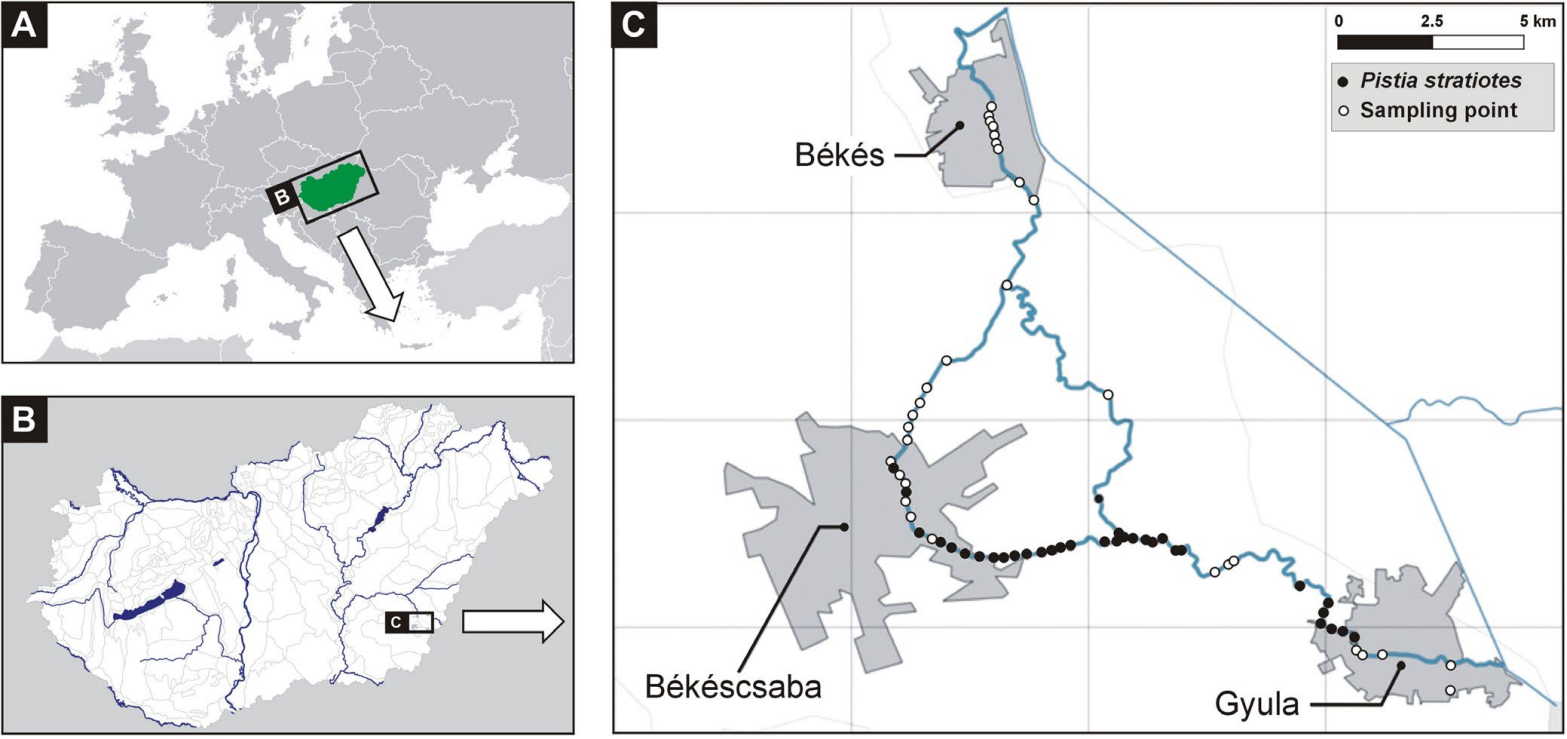
Distribution of *Pistia stratiotes* along the Élővíz Canal (Élővíz‐csatorna) near Békéscsaba and Gyula, southeastern Hungary.

The distribution of *Pistia stratiotes* was surveyed in September 2022 along the Élővíz‐csatorna (Élővíz Canal) and its tributaries within the administrative areas of the towns of Békés, Békéscsaba, and Gyula in south‐eastern Hungary. The width of the canal is relatively uniform, ranging between approximately 8 and 10 m along its entire length. In total, 71 sampling points were established along the watercourse (Figure 1). Each sampling point comprised an approximately 50 m long canal section. Consecutive points were placed at intervals of typically 500 m, occasionally ranging between 300 and 900 m, depending on accessibility and visibility as determined by the surrounding environment and riparian vegetation. At each sampling point, the presence or absence of *Pistia stratiotes* , its coverage (m^2^), and the presence of flowering individuals were recorded. All sampling locations were revisited on 13 January 2023 to assess overwinter survival; no floating individuals were detected. However, the presence of submerged propagules (e.g., seeds or overwintering vegetative structures) cannot be excluded.

Water chemistry (pH, electrical conductivity, water temperature, and dissolved oxygen concentration) was measured on 13 September 2022 at 11 localities, including both occupied (*n* = 7) and unoccupied (*n* = 4) sites, using a Hach HQ40D multimeter.

Additional measurements were carried out in January 2023 at 26 localities, including sites previously occupied in September 2022 (*n* = 16) as well as previously unoccupied sites (*n* = 10). These winter measurements were used to characterise seasonal environmental conditions and were not included in formal statistical comparisons because of differences in seasonal context. The sampling sites were selected to represent different sections of the canal system.

Water velocity and the number of drifting individuals transported by the current were recorded following the methodology described by Kis and Molnár (2026). Drifting individuals were quantified as the number of rosettes passing a fixed observation point during a 10‐min interval.

To assess the critical size associated with flowering and the extent of vegetative reproduction, maximum leaf length was measured, and the number of inflorescences and vegetative ramets was recorded for 126 randomly selected individuals collected from five localities (Table S2). These sites were selected to represent different population sizes, and sampling was conducted proportionally to local population density. The number of flowering individuals reflects their low proportion within the population. Maximum leaf length was used as a proxy for plant size, as it can be measured reliably in the field and has been shown to correlate with biomass and reproductive output in *Pistia stratiotes* (Freitas Coelho et al. 2005). The locations used for water chemistry measurements, flow measurements, and trait sampling were not necessarily the same.

### 2.2 Data Analysis

Differences in maximum leaf length between vegetative and flowering individuals were evaluated using the Mann–Whitney *U*‐test, because the data did not meet the assumptions of normality and sample sizes were highly unequal. Differences in the number of ramets between vegetative and flowering individuals were analysed using a Mann–Whitney *U*‐test, as ramet counts were discrete and highly skewed and sample sizes differed substantially. All statistical analyses were performed using non‐parametric tests to account for these distributional properties using Jamovi version 2.7.6 (The Jamovi Project 2025; R Core Team 2025).

## 3 Results

### 3.1 Occurrence and Spatial Pattern

**FIGURE 2:**
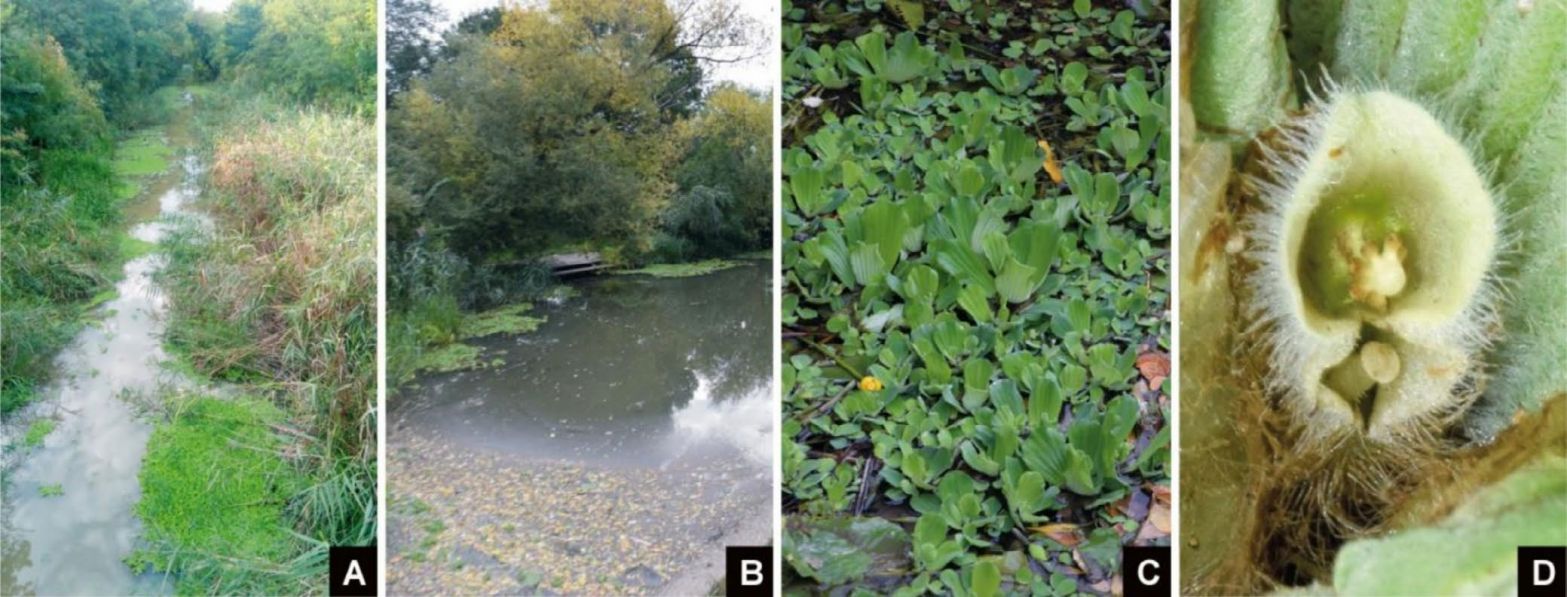
*Pistia stratiotes* in Élővíz Canal (Békés county, SE‐Hungary). A & B–habitats, C–mass occurrence. D–inflorescence.

*Pistia stratiotes* was detected at 35 of the 71 surveyed sampling points (49.3%) in the vicinity of Békéscsaba and Gyula (Figure 2), covering a total area of 141 m^2^. Approximately 77% (27 of 35) of the recorded occurrences consisted of small patches (< 3 m^2^), but locally the species formed extensive mats, with a maximum cover of 60 m^2^ at the largest site (Table 1).

**TABLE 1:** Occurrences of *Pistia stratiotes* along canals near Békéscsaba and Gyula (Southeast‐Hungary).

ID	Settlement	Locality	Geocoordinates	Cover of *Pistia stratiotes* (m^2^)	Presence of flowering *Pistia stratiotes*
ÉV‐21	Békéscsaba	Élővíz Canal	46.689451° N 21.096324° E	0.02	
ÉV‐24	Békéscsaba	Élővíz Canal	46.683617° N 21.100593° E	0.04	
ÉV‐27	Békéscsaba	Élővíz Canal	46.673820° N 21.105385° E	0.5	
ÉV‐28	Békéscsaba	Élővíz Canal	46.672490° N 21.109643° E	1	
ÉV‐29	Békéscsaba	Élővíz Canal	46.671497° N 21.112993° E	60	
ÉV‐30	Békéscsaba	Élővíz Canal	46.670388° N 21.116675° E	15	
ÉV‐31	Békéscsaba	Élővíz Canal	46.668800° N 21.121418° E	3	
ÉV‐32	Békéscsaba	Élővíz Canal	46.668155° N 21.126580° E	3	+
ÉV‐33	Békéscsaba	Élővíz Canal	46.667940° N 21.131697° E	0.5	
ÉV‐34	Békéscsaba	Élővíz Canal	46.667930° N 21.135192° E	2.5	
ÉV‐35	Békéscsaba	Élővíz Canal	46.668200° N 21.138897° E	1	+
ÉV‐36	Békéscsaba	Élővíz Canal	46.668664° N 21.143287° E	1	
ÉV‐37	Békéscsaba	Élővíz Canal	46.669162° N 21.148444° E	1.5	
ÉV‐38	Békéscsaba	Élővíz Canal	46.669689° N 21.152422° E	2	+
ÉV‐39	Békéscsaba	Élővíz Canal	46.670302° N 21.154912° E	3	
ÉV‐40	Békéscsaba	Élővíz Canal	46.670706° N 21.158425° E	1.5	+
ÉV‐42	Békéscsaba	Élővíz Canal	46.671634° N 21.170659° E	1.5	
ÉV‐41	Békéscsaba	Élővíz Canal	46.672013° N 21.174860° E	2	+
GE‐01	Békéscsaba	Gerlai‐holtág	46.672778° N 21.176994° E	10	+
GE‐03	Békéscsaba	Gerlai‐holtág	46.673667° N 21.175626° E	20	
KF‐01	Békéscsaba	Kígyósi‐főcs.	46.672931° N 21.177226° E	0.5	
GE‐04	Békéscsaba	Gerlai‐holtág	46.682076° N 21.168427° E	0.5	
ÉV‐48	Békéscsaba	Élővíz Canal	46.672448° N 21.180426° E	0.25	
ÉV‐49	Békéscsaba	Élővíz Canal	46.672077° N 21.185103° E	3	
ÉV‐50	Békéscsaba	Élővíz Canal	46.671534° N 21.187340° E	1	
ÉV‐51	Békéscsaba	Élővíz Canal	46.672434° N 21.190904° E	1	
ÉV‐53	Békéscsaba	Élővíz Canal	46.669689° N 21.196024° E	1.5	
ÉV‐52	Békéscsaba	Élővíz Canal	46.669735° N 21.197449° E	1	
ÉV‐57	Gyula	Élővíz Canal	46.661199° N 21.239212° E	0.1	
ÉV‐58	Gyula	Élővíz Canal	46.656845° N 21.248987° E	0.2	
ÉV‐59	Gyula	Élővíz Canal	46.654687° N 21.247535° E	0.04	
ÉV‐60	Gyula	Élővíz Canal	46.652020° N 21.246642° E	0.1	
ÉV‐61	Gyula	Élővíz Canal	46.650601° N 21.250414° E	1	
ÉV‐62	Gyula	Élővíz Canal	46.650063° N 21.254366° E	0.08	
ÉV‐64	Gyula	Élővíz Canal	46.648598° N 21.258439° E	2	

### 3.2 Water Chemistry

Localities where *Pistia stratiotes* was recorded were characterised by strongly alkaline waters (Table 2.), with pH values ranging from 9.28 to 9.69; at 5 out of 11 sampling points, pH exceeded 9.50. Electrical conductivity ranged between 320 and 434 μS cm^−1^ (mean ± SD: 388 ± 35 μS cm^−1^), indicating low to moderate mineral content typical of non‐saline canal waters. Water temperature at the time of September sampling ranged from 15.6°C to 21.1°C. Dissolved oxygen concentrations were similar across 10 of the 11 sampling points (mean ± SD: 6.79 ± 0.92 mg L^−1^), whereas one locality exhibited a markedly higher value (22 mg L^−1^), likely because of the presence of filamentous algae.

**TABLE 2:** Water chemistry parameters along the canal system of Élővíz‐csatorna. The numbering of localities corresponds to that used in Table 1 and Table S1.

ID	Date	pH	Conductivity (μS/cm2)	Temperature (°C)	O_2_ content (mg/L)	Presence of *Pistia* in September 2022
ÉV‐02	13/09/2022	9.29	408	14.7	5.9	−
ÉV‐10	13/09/2022	9.36	431	16.0	5.47	−
ÉV‐11	13/09/2022	9.42	415	15.7	6.28	−
ÉV‐13	13/09/2022	9.69	348	16.8	8.61	−
ÉV‐21	13/09/2022	9.5	374	18.5	6.46	+
ÉV‐28	13/09/2022	9.49	372	17.6	6.66	+
ÉV‐29	13/09/2022	9.56	371	17.7	7.22	+
ÉV‐49	13/09/2022	9.69	409	16.6	7.33	+
ÉV‐53	13/09/2022	9.59	390	17.2	7.60	+
ÉV‐59	13/09/2022	9.37	432	21.1	6.32	+
KF‐01	13/09/2022	11.28	320	16.9	22.00	+
ÉV‐02	13/01/2023	7.86	532	8.0	7.25	−
ÉV‐03	13/01/2023	7.83	529	7.40	7.06	−
ÉV‐06	13/01/2023	7.84	515	7.4	7.16	−
ÉV‐07	13/01/2023	7.88	507	7.5	7.10	−
ÉV‐08	13/01/2023	7.9	502	7.4	7.60	−
ÉV‐09	13/01/2023	7.9	492	7.2	7.45	−
ÉV‐10	13/01/2023	7.97	480	7.4	8.97	−
ÉV‐11	13/01/2023	7.94	478	7.4	8.63	−
ÉV‐13	13/01/2023	8.13	290	6.6	10.4	−
ÉV‐21	13/01/2023	8.03	429	7.9	8.41	+
ÉV‐24	13/01/2023	8.09	416	7.8	8.36	+
ÉV‐25	13/01/2023	8.38	611	8.0	9.65	−
ÉV‐31	13/01/2023	8.78	693	6.6	11.65	+
ÉV‐32	13/01/2023	8.68	684	6.7	11.65	+
GE‐03	13/01/2023	8.4	713	6.7	9.88	+
ÉV‐41	13/01/2023	8.44	653	6.8	9.76	+
KF‐01	13/01/2023	8.33	845	6.5	9.14	+
ÉV‐49	13/01/2023	8.41	554	6.6	10.18	+
ÉV‐50	13/01/2023	8.38	491	6.7	10.06	+
ÉV‐51	13/01/2023	8.27	436	6.7	9.64	+
ÉV‐58	13/01/2023	8.18	439	6.9	8.95	+
ÉV‐59	13/01/2023	8.46	444	6.7	9.04	+
ÉV‐60	13/01/2023	8.09	461	7.1	8.12	+
ÉV‐61	13/01/2023	8.12	494	7.0	8.07	+
ÉV‐62	13/01/2023	8.13	523	7.0	7.89	+
ÉV‐64	13/01/2023	8.2	524	6.9	8.12	+

*Note:* The presence of *Pistia* refers to site identity/September occurrence, not observed floating individuals in January.

In January 2023, water chemistry conditions differed markedly from those recorded in September. pH values were substantially lower, ranging from 7.83 to 8.78, indicating neutral to moderately alkaline conditions. Electrical conductivity generally increased and showed a wider range (290–845 μS cm^−1^), with several sites exhibiting considerably higher values than those measured in early autumn. Water temperature decreased to 6.5°C–8.0°C across all sampling points, reflecting winter conditions. Dissolved oxygen concentrations were generally higher and less variable than in September, ranging from 7.06 to 11.65 mg L^−1^.

### 3.3 Water Velocity and Drift

Localities of *Pistia stratiotes* were associated with slow‐flowing canal sections, where water velocity ranged from 0.05 to 0.32 m s^−1^ (Table 3). 57.1% of the study sites exhibited low current velocities (≤ 0.16 m s^−1^), reflecting lentic to weakly lotic conditions typical of managed lowland canals. The number of drifting *Pistia* individuals recorded over 10‐min intervals varied widely, from 2 to 73 individuals, indicating substantial spatial differences in propagule transport (mean ± SD: 30 ± 37 individuals per 10‐min interval). Although a positive relationship was observed between water velocity and the number of drifting rosettes, this was not statistically significant (linear regression: *p* = 0.081; Spearman's ρ = 0.286, *p* = 0.556).

**TABLE 3:** Velocity of water (m/s) and number of drifting *Pistia stratiotes* individuals/10 min.

ID	Settlement	Locality	Velocity of water (m/s)	Number of drifting *Pistia‐*individuals/10 min
ÉV‐30	Békéscsaba	Élővíz Canal	0.1	13
ÉV‐31	Békéscsaba	Élővíz Canal	0.12	5
ÉV‐32	Békéscsaba	Élővíz Canal	0.16	7
ÉV‐41	Békéscsaba	Élővíz Canal	0.05	4
ÉV‐53	Békéscsaba	Élővíz Canal	0.32	73
ÉV‐56	Békéscsaba	Élővíz Canal	0.19	2
ÉV‐59	Gyula	Élővíz Canal	0.14	52

*Note:* The numbering of localities is the same as in Table 1.

### 3.4 Flowering and Vegetative Reproduction

**FIGURE 3:**
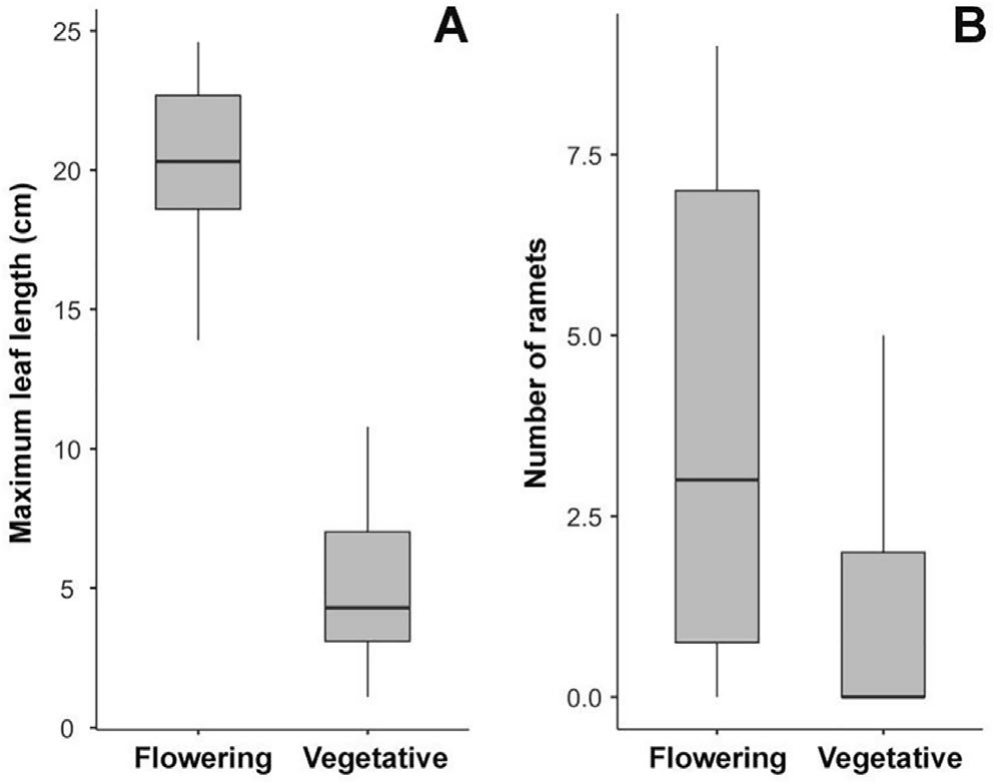
Comparison of flowering and vegetative individuals of *Pistia stratiotes* . A. *maximum* leaf length (cm). B. Number of ramets per individual. Differences between groups were statistically significant for both variables (Mann–Whitney *U*‐test: *p* < 0.001 for leaf length; *p* = 0.002 for number of ramets).

Flowering individuals were recorded at six localities (Table 1). Of the 126 measured individuals, 16 (12.7%) were flowering, bearing 1–3 inflorescences per individual (mean ± SD = 1.69 ± 0.70). Flowering individuals had markedly larger maximum leaf lengths (approximately fourfold higher mean values) and produced more ramets than vegetative ones (Figure 3A,B; Table 4). Both differences were statistically significant, indicating a clear size threshold for flowering and a concomitant increase in clonal propagation among reproductive individuals.

**TABLE 4:** Comparison of generative and vegetative individuals of *Pistia stratiotes* : Group descriptives for statistical analysis and results of the Mann–Whitney *U*‐test made by Jamovi.

Variable	Group	n	Mean	Median	SD	SE	*U* value	*p*
Maximum leaf length (cm)	Flowering	16	20.23	20.30	3.08	0.769	4.00	< 0.001
	Vegetative	110	5.27	4.30	3.07	0.293		
Number of ramets	Flowering	16	6.69	6.00	5.99	1.496	511.00	0.006
	Vegetative	110	3.19	2.00	3.07	0.294		

*Note:* H_a_ μ Flowering ≠ μ Vegetative.

## 4 Discussion

### 4.1 Thermally Modified Microrefugia and Climatic Constraints

Our findings support the emerging view that thermally modified freshwater systems can function as stepping‐stone habitats enabling warm‐adapted invaders to cross formerly prohibitive climatic barriers. Such microrefugia may therefore accelerate poleward range expansion under ongoing climate warming, even when thermal anomalies are spatially limited or intermittent.

The mean water temperature of 17.16°C ± 1.67°C recorded in early autumn was favourable for the flowering of floating macrophytes, although this was lower than the values measured in the thermal streams sustaining perennial populations in Slovenia, which remained above 17°C throughout the year (Šajna et al. 2007).

However, winter measurements revealed substantially lower temperatures (6.5°C–8.0°C), indicating that the studied canal system does not represent a truly thermal environment but rather a seasonally buffered system with partial thermal influence. Here, partial thermal influence refers to a consistent elevation of water temperature relative to ambient conditions without reaching fully thermal regimes. Such conditions may be sufficient to sustain viable populations, even in the absence of persistently high temperatures. This is particularly likely when combined with sheltered hydrological conditions that reduce mechanical disturbance and heat loss. These observations are also consistent with earlier expectations regarding the climatic limitations of the species in Hungary. Although Danyik and Vidéki (2012) predicted that mass occurrences of *P. stratiotes* in Hungary were unlikely because of its cold sensitivity and severe winter periods, the species has persisted for more than a decade in the Békéscsaba area. This longevity underscores the significance of thermally abnormal waters as refugia for alien macrophytes and raises the possibility that such habitats may promote the selection of more cold‐tolerant lineages, potentially enhancing the species' capacity for broader regional spread. However, this possibility remains speculative and cannot be inferred from the present data and would require dedicated physiological or genetic studies to verify. Ongoing climate change, particularly the reduction of cold‐temperature extremes (Bartholy et al. 2014), may further enhance the ecological feasibility of persistence in temperate regions. However, long‐term establishment should primarily be interpreted here as environmentally mediated persistence rather than evidence of adaptive range expansion.

Numerous studies indicate that elevated temperatures can enhance growth and physiological performance in tropical and non‐native aquatic macrophytes (Šajna et al. 2023; Wang et al. 2024; Xiang et al. 2025). However, direct evidence for selection or adaptive evolutionary responses in thermally influenced freshwater systems remains limited. Some studies have demonstrated population‐level differences in physiological responses to temperature, for example, in *Ludwigia peploides* (Thiébaut et al. 2021), suggesting the potential for local differentiation. Nevertheless, such processes cannot be inferred from the present data and require targeted experimental and genetic investigations.

### 4.2 Habitat Association and Environmental Conditions

Our results demonstrate that *Pistia stratiotes* populations along the Élővíz Canal are associated with slow‐flowing canal sections. These habitats are characterised by alkaline waters and moderate mineralisation. Although Danyik and Vidéki (2012) reported that the species prefers slightly acidic waters (pH 6.5–7.2), the consistently high pH values recorded across our survey localities fall within the tolerance range documented for the species in both its native and invaded regions (Galal et al. 2019; Sbaghi and El Aalaoui 2024). Conductivity values likewise indicate that *P. stratiotes* can successfully colonise lowland canals with relatively low ionic content, consistent with observations from Central European and Mediterranean waters (Jaklič et al. 2020; Šajna et al. 2023). It should be noted that the studied canal system is characterised by a low abundance of native macrophytes, which limited the possibility of assessing interactions between native and non‐native species. Such interactions in thermally influenced systems have been addressed in previous studies (e.g., Koleszár, Lukács, et al. 2024; Koleszár, Szabó, et al. 2024).

### 4.3 Hydrological Control of Dispersal

Our results highlight the dual role of hydrology in shaping both local persistence and downstream spread. Occupied localities were consistently associated with low flow velocities, supporting previous findings that lentic or weakly lotic environments facilitate mat formation and biomass accumulation (Hall and Okali 1974; Milićević 2023). In contrast, episodic increases in flow velocity led to pronounced increases in drifting individuals, indicating that flow‐mediated transport constitutes an efficient dispersal mechanism within canal networks.

This duality—local retention under low‐flow conditions and long‐distance dispersal during high‐flow events—has important implications for invasion dynamics. Similar patterns have been documented in other artificial waterways, where managed discharge regimes unintentionally enhance downstream propagule pressure (Kis and Molnár 2026). In the Élővíz Canal, such processes likely contribute to the mosaic distribution of *P. stratiotes* , with dense patches interspersed among sparsely colonised sections. Subtle variations in canal management can, therefore, substantially reshape invasion trajectories, consistent with conceptual models emphasising disturbance pulses and propagule transport in linear aquatic corridors.

### 4.4 Reproductive Performance at the Invasion Front

Despite its primarily vegetative mode of reproduction, *P. stratiotes* exhibited regular flowering in the study area. Flowering individuals represented 11% of sampled plants, comparable to proportions reported in other temperate invaded regions (Borsic and Rubinić 2021) but lower than values observed in tropical populations (Freitas Coelho et al. 2005). The clear size threshold associated with flowering, expressed in significantly larger leaf sizes of reproductive individuals, aligns with previous findings on size‐dependent reproductive allocation (Freitas Coelho et al. 2005; Pieterse et al. 1981). For example, Freitas Coelho et al. (2005) reported a significant positive relationship between rosette leaf size and the biomass of reproductive structures in populations from the species' native range, indicating that size‐dependent reproductive allocation is a consistent trait across different environmental contexts. However, the size threshold for flowering observed in our study appears considerably higher than that reported from the species' native range. Freitas Coelho et al. (2005) found that flowering may occur at leaf lengths exceeding 2.8 cm under favourable conditions, whereas in our study, the smallest flowering individual measured 13.9 cm (mean ± SD: 20.3 ± 3.1 cm). This suggests that reproductive thresholds may shift under temperate conditions, potentially reflecting environmental constraints at the climatic margin.

Notably, flowering individuals produced significantly more vegetative ramets, indicating that reproductive individuals also exhibit increased clonal propagation, which is likely associated with plant size rather than directly reflecting local environmental conditions. This pattern likely reflects size‐dependent allocation to both sexual and clonal reproduction. However, without information on seed viability and recruitment, the demographic consequences of sexual reproduction at the invasion front remain uncertain. Such trait‐based amplification mechanisms have been recognised as key drivers of establishment success in warm‐adapted macrophytes entering temperate ecosystems (Koleszár, Lukács, et al. 2024; Koleszár, Szabó, et al. 2024).

### 4.5 Introduction Pathways and Human‐Mediated Spread

Although Lukács et al. (2016) classified *Pistia stratiotes* as belonging to the category of occurrences arising without human assistance, mediated by natural dispersal agents, Kaplan et al. (2016) argued that the increasing frequency of records of conspicuous alien aquatic plants in the Czech Republic over the past 25 years—such as *Eichhornia crassipes* , *P. stratiotes,* and *Pontederia cordata* —is primarily attributable to their deliberate introduction, not only into garden ponds but also into natural wetland habitats. This latter pathway, together with the species' considerable horticultural and ornamental appeal, is supported by the fact that *P. stratiotes* remains readily available from Hungarian horticultural suppliers and online shops (see Web sources [2–5]). Csiky et al. (2023) likewise classified the species in the *deliberate* category. According to Glaser et al. (2025), the species spreads both through escape and stowaway pathways. These considerations suggest that human‐mediated propagule pressure likely played a role in the establishment of the species in the Élővíz Canal, complementing the environmental mechanisms discussed above.

This study has several limitations that should be considered when interpreting the results. Water chemistry was measured at a limited number of sampling points, which constrains conclusions regarding environmental homogeneity across the canal system.

Although both occupied and unoccupied sites were included, the limited number of sampling points precluded a robust comparison of abiotic conditions between presence and absence locations. Winter measurements were also available; however, differences in sampling design, spatial coverage, and temporal resolution prevented formal seasonal comparisons. Consequently, the observed seasonal patterns should be interpreted descriptively. Reproductive analyses were based on a relatively small number of flowering individuals, and seed viability was not assessed, highlighting the need for further investigation. In addition, the absence of comparative data from non‐invaded or non‐thermal systems limits broader ecological generalisations.

### 4.6 Implications for Invasion Risk Under Climate Change

The establishment and apparent multi‐year persistence of *Pistia stratiotes* in the Élővíz Canal highlight the growing invasion risk posed by warm‐adapted aquatic macrophytes in Central Europe. Climate change is likely to further reduce winter mortality and expand the range of thermally suitable habitats, particularly in anthropogenically warmed systems (Šajna et al. 2023). Even in the absence of persistent thermal input, increased physiological plasticity and shading tolerance may facilitate survival under increasingly variable conditions (Koleszár, Lukács, et al. 2024; Koleszár, Szabó, et al. 2024).

Given its demonstrated ability to outcompete native macrophytes and alter ecological conditions (Jaklič et al. 2020), the continued spread of *P. stratiotes* in Hungarian canal systems warrants close monitoring. Overall, our results should be interpreted as evidence of ecological persistence under thermally influenced conditions rather than of demonstrated evolutionary change. From a management perspective, our results highlight the importance of monitoring thermally influenced canal sections and managing flow regimes where possible to limit downstream dispersal. Early detection and targeted removal in source areas may help reduce further spread within canal networks. In practical terms, management efforts should focus on early detection and rapid removal of newly established populations, particularly in upstream or thermally buffered sections that may function as source areas.

## Author Contributions

**Szabolcs Kis:** investigation (equal), methodology (equal), visualization (equal), writing – original draft (equal), writing – review and editing (equal). **Attila Molnár V.:** conceptualization (lead), formal analysis (equal), investigation (equal), methodology (equal), visualization (equal), writing – original draft (equal), writing – review and editing (equal).

## Funding

This work was supported by Debreceni Egyetem, EKÖP‐25‐3‐I‐DE‐367. National Research, Development and Innovation Office (Hungary), MEC N 24 148930, OTKA K132573. Count István Tisza Foundation for the University of Debrecen, PhD Excellence Scholarship.

## Conflicts of Interest

The authors declare no conflicts of interest.

## Supporting information


**Table S1:** Sampling locations along Élővíz Canal at which the presence of *Pistia stratiotes* was not recorded.
**Table S2:** Morphological traits of *Pistia stratiotes* measured on Élővíz Canal. Note: the numbering of localities corresponds to that used in Table 1.

## Data Availability

All data used in the preparation of this manuscript are fully included in the tables.
