# Supplementary material for: Data on the Distribution and Ecology of Pistia stratiotes L. (Araceae) in Hungary
Source: Ecol Evol. 2026 Apr 30;16(5):e73488. doi: 10.1002/ece3.73488 (PMC13130341; doi:10.1002/ece3.73488)
Supplement: Supplementary file 1 — Table S1: Sampling locations along Élővíz Canal at which the presence of Pistia stratiotes was not recorded. Table S2: Morphological traits of Pistia stratiotes measured on Élővíz Canal. Note: the numbering of localities corresponds to that used in Table 1. [file ECE3-16-e73488-s001.docx]

Supplementary material

**Table S1.** Sampling locations along Élővíz Canal at which presence of *Pistia stratiotes* was not recorded.

| ID | Settlement | Locality | Geocoordinates |
| --- | --- | --- | --- |
| ÉV-01 | Békés | Élővíz-csatorna | 46.776565° N 21.130665° E |
| ÉV-02 | Békés | Élővíz-csatorna | 46.774325° N 21.129658° E |
| ÉV-04 | Békés | Élővíz-csatorna | 46.772988° N 21.130259° E |
| ÉV-03 | Békés | Élővíz-csatorna | 46.772330° N 21.130905° E |
| ÉV-05 | Békés | Élővíz-csatorna | 46.771404° N 21.131542° E |
| ÉV-06 | Békés | Élővíz-csatorna | 46.770613° N 21.131657° E |
| ÉV-07 | Békés | Élővíz-csatorna | 46.769679° N 21.131851° E |
| ÉV-08 | Békés | Élővíz-csatorna | 46.767790° N 21.132469° E |
| ÉV-09 | Békés | Élővíz-csatorna | 46.766431° N 21.133063° E |
| ÉV-10 | Békés | Élővíz-csatorna | 46.758339° N 21.140693° E |
| ÉV-11 | Békés | Élővíz-csatorna | 46.753977° N 21.145629° E |
| ÉV-13 | Békéscsaba | Élővíz-csatorna | 46.733833° N 21.136120° E |
| ÉV-12 | Békéscsaba | Élővíz-csatorna | 46.733397° N 21.135666° E |
| GE-01 | Békéscsaba | Gerlai-holtág | 46.733556° N 21.136528° E |
| ÉV-18 | Békéscsaba | Élővíz-csatorna | 46.715478° N 21.115240° E |
| ÉV-17 | Békéscsaba | Élővíz-csatorna | 46.709004° N 21.108344° E |
| ÉV-16 | Békéscsaba | Élővíz-csatorna | 46.705339° N 21.105622° E |
| ÉV-15 | Békéscsaba | Élővíz-csatorna | 46.701986° N 21.102874° E |
| ÉV-14 | Békéscsaba | Élővíz-csatorna | 46.699252° N 21.101687° E |
| ÉV-20 | Békéscsaba | Élővíz-csatorna | 46.696333° N 21.101311° E |
| ÉV-19 | Békéscsaba | Élővíz-csatorna | 46.690818° N 21.095770° E |
| ÉV-22 | Békéscsaba | Élővíz-csatorna | 46.687850° N 21.098495° E |
| ÉV-23 | Békéscsaba | Élővíz-csatorna | 46.685925° N 21.100612° E |
| ÉV-26 | Békéscsaba | Élővíz-csatorna | 46.681599° N 21.100618° E |
| ÉV-25 | Békéscsaba | Élővíz-csatorna | 46.677781° N 21.102071° E |
| GE-05 | Békéscsaba | Élővíz-csatorna | 46.707287° N 21.171722° E |
| ÉV-56 | Békéscsaba | Élővíz-csatorna | 46.664346° N 21.209221° E |
| ÉV-55 | Békéscsaba | Élővíz-csatorna | 46.666205° N 21.214005° E |
| ÉV-54 | Békéscsaba | Élővíz-csatorna | 46.666933° N 21.216016° E |
| ÉV-63 | Gyula | Élővíz-csatorna | 46.649159° N 21.258204° E |
| ÉV-65 | Gyula | Élővíz-csatorna | 46.647805° N 21.258114° E |
| ÉV-66 | Gyula | Élővíz-csatorna | 46.645401° N 21.259243° E |
| ÉV-67 | Gyula | Élővíz-csatorna | 46.644390° N 21.261272° E |
| ÉV-68 | Gyula | Élővíz-csatorna | 46.644457° N 21.268069° E |
| ÉV-69 | Gyula | Élővíz-csatorna | 46.642088° N 21.292021° E |
| ÉV-65 | Gyula | Élővíz-csatorna | 46.635716° N 21.291979° E |

**Table S2.** Morphological traits of *Pistia stratiotes* measured on Élővíz Canal. Note: the numbering of localities corresponds to that used in Table 1.

| ID | Maximum leaf length (cm) | Number of inflorescences | Number of ramets | Status |
| --- | --- | --- | --- | --- |
| ÉV-29 | 15.5 | 2 | 2 | Flowering |
| ÉV-29 | 23.9 | 1 | 8 | Flowering |
| ÉV-29 | 23.6 | 2 | 7 | Flowering |
| ÉV-29 | 18.7 | 1 | 2 | Flowering |
| ÉV-29 | 13.9 | 1 | 10 | Flowering |
| ÉV-29 | 18.3 | 2 | 7 | Flowering |
| ÉV-29 | 20.8 | 1 | 15 | Flowering |
| ÉV-29 | 24.6 | 2 | 3 | Flowering |
| ÉV-29 | 9.8 | 0 | 9 | Vegetative |
| ÉV-29 | 7.1 | 0 | 4 | Vegetative |
| ÉV-29 | 3.8 | 0 | 1 | Vegetative |
| ÉV-29 | 6.4 | 0 | 4 | Vegetative |
| ÉV-29 | 5.7 | 0 | 2 | Vegetative |
| ÉV-29 | 10.0 | 0 | 8 | Vegetative |
| ÉV-29 | 5.8 | 0 | 10 | Vegetative |
| ÉV-29 | 7.3 | 0 | 8 | Vegetative |
| ÉV-29 | 18.1 | 0 | 4 | Vegetative |
| ÉV-29 | 8.7 | 0 | 6 | Vegetative |
| ÉV-29 | 10.8 | 0 | 3 | Vegetative |
| ÉV-29 | 5.6 | 0 | 4 | Vegetative |
| ÉV-29 | 13.4 | 0 | 2 | Vegetative |
| ÉV-29 | 9.4 | 0 | 10 | Vegetative |
| ÉV-29 | 7.1 | 0 | 11 | Vegetative |
| ÉV-29 | 5.5 | 0 | 3 | Vegetative |
| ÉV-29 | 4.8 | 0 | 0 | Vegetative |
| ÉV-29 | 3.4 | 0 | 3 | Vegetative |
| ÉV-29 | 3.3 | 0 | 0 | Vegetative |
| ÉV-29 | 14.6 | 0 | 1 | Vegetative |
| ÉV-29 | 8.6 | 0 | 8 | Vegetative |
| ÉV-29 | 5.2 | 0 | 14 | Vegetative |
| ÉV-29 | 10.6 | 0 | 2 | Vegetative |
| ÉV-29 | 6.6 | 0 | 3 | Vegetative |
| ÉV-29 | 1.1 | 0 | 3 | Vegetative |
| ÉV-29 | 4.6 | 0 | 2 | Vegetative |
| ÉV-29 | 2.0 | 0 | 0 | Vegetative |
| ÉV-29 | 2.8 | 0 | 2 | Vegetative |
| ÉV-29 | 3.8 | 0 | 5 | Vegetative |
| ÉV-29 | 3.5 | 0 | 2 | Vegetative |
| ÉV-29 | 2.7 | 0 | 5 | Vegetative |
| ÉV-29 | 4.4 | 0 | 2 | Vegetative |
| ÉV-29 | 10.2 | 0 | 5 | Vegetative |
| ÉV-29 | 4.3 | 0 | 2 | Vegetative |
| ÉV-29 | 4.7 | 0 | 5 | Vegetative |
| ÉV-29 | 2.1 | 0 | 3 | Vegetative |
| ÉV-29 | 2.5 | 0 | 2 | Vegetative |
| ÉV-29 | 2.0 | 0 | 2 | Vegetative |
| ÉV-29 | 2.5 | 0 | 2 | Vegetative |
| ÉV-29 | 2.0 | 0 | 2 | Vegetative |
| ÉV-29 | 2.2 | 0 | 2 | Vegetative |
| ÉV-29 | 3.1 | 0 | 2 | Vegetative |
| ÉV-29 | 2.8 | 0 | 1 | Vegetative |
| ÉV-29 | 5.2 | 0 | 3 | Vegetative |
| ÉV-29 | 7.8 | 0 | 4 | Vegetative |
| ÉV-29 | 4.1 | 0 | 3 | Vegetative |
| ÉV-29 | 8.5 | 0 | 3 | Vegetative |
| ÉV-29 | 4.3 | 0 | 2 | Vegetative |
| ÉV-29 | 6.1 | 0 | 5 | Vegetative |
| ÉV-29 | 10.2 | 0 | 15 | Vegetative |
| ÉV-29 | 10.4 | 0 | 3 | Vegetative |
| ÉV-29 | 7.4 | 0 | 7 | Vegetative |
| ÉV-29 | 7.9 | 0 | 6 | Vegetative |
| ÉV-29 | 3.2 | 0 | 1 | Vegetative |
| ÉV-29 | 3.5 | 0 | 3 | Vegetative |
| ÉV-29 | 4.2 | 0 | 5 | Vegetative |
| ÉV-29 | 4.9 | 0 | 2 | Vegetative |
| ÉV-29 | 8.3 | 0 | 0 | Vegetative |
| ÉV-29 | 5.6 | 0 | 4 | Vegetative |
| ÉV-29 | 7.6 | 0 | 6 | Vegetative |
| ÉV-29 | 4.1 | 0 | 2 | Vegetative |
| ÉV-29 | 6.8 | 0 | 2 | Vegetative |
| ÉV-29 | 5.0 | 0 | 1 | Vegetative |
| ÉV-29 | 6.2 | 0 | 0 | Vegetative |
| ÉV-29 | 3.3 | 0 | 0 | Vegetative |
| ÉV-29 | 3.2 | 0 | 1 | Vegetative |
| ÉV-29 | 1.7 | 0 | 0 | Vegetative |
| ÉV-29 | 2.8 | 0 | 1 | Vegetative |
| ÉV-29 | 3.0 | 0 | 3 | Vegetative |
| ÉV-29 | 2.2 | 0 | 0 | Vegetative |
| ÉV-29 | 4.5 | 0 | 3 | Vegetative |
| ÉV-29 | 1.9 | 0 | 0 | Vegetative |
| ÉV-29 | 5.1 | 0 | 0 | Vegetative |
| ÉV-29 | 1.9 | 0 | 0 | Vegetative |
| ÉV-29 | 4.8 | 0 | 2 | Vegetative |
| ÉV-29 | 3.1 | 0 | 3 | Vegetative |
| ÉV-29 | 4.3 | 0 | 10 | Vegetative |
| ÉV-29 | 6.1 | 0 | 11 | Vegetative |
| ÉV-29 | 2.9 | 0 | 3 | Vegetative |
| ÉV-29 | 3.6 | 0 | 1 | Vegetative |
| ÉV-29 | 4.2 | 0 | 2 | Vegetative |
| ÉV-29 | 3.1 | 0 | 3 | Vegetative |
| ÉV-29 | 3.5 | 0 | 1 | Vegetative |
| ÉV-29 | 3.7 | 0 | 5 | Vegetative |
| ÉV-29 | 4.4 | 0 | 1 | Vegetative |
| ÉV-29 | 4.2 | 0 | 3 | Vegetative |
| ÉV-29 | 6.2 | 0 | 7 | Vegetative |
| ÉV-29 | 3.6 | 0 | 3 | Vegetative |
| ÉV-29 | 2.5 | 0 | 1 | Vegetative |
| ÉV-29 | 6.3 | 0 | 3 | Vegetative |
| ÉV-29 | 3.4 | 0 | 1 | Vegetative |
| ÉV-32 | 23.2 | 1 | 1 | Flowering |
| ÉV-32 | 17.2 | 3 | 0 | Flowering |
| ÉV-32 | 19.8 | 1 | 3 | Flowering |
| ÉV-32 | 22.1 | 2 | 8 | Flowering |
| ÉV-32 | 3.4 | 0 | 2 | Vegetative |
| ÉV-32 | 9.5 | 0 | 9 | Vegetative |
| ÉV-32 | 2.2 | 0 | 0 | Vegetative |
| ÉV-32 | 3.2 | 0 | 3 | Vegetative |
| ÉV-32 | 3.2 | 0 | 1 | Vegetative |
| ÉV-32 | 7.8 | 0 | 5 | Vegetative |
| ÉV-32 | 2.4 | 0 | 0 | Vegetative |
| ÉV-32 | 7.9 | 0 | 5 | Vegetative |
| ÉV-32 | 2.5 | 0 | 1 | Vegetative |
| ÉV-32 | 4.2 | 0 | 2 | Vegetative |
| ÉV-32 | 2.0 | 0 | 1 | Vegetative |
| ÉV-38 | 22.5 | 2 | 5 | Flowering |
| ÉV-38 | 19.6 | 3 | 5 | Flowering |
| ÉV-38 | 2.4 | 0 | 0 | Vegetative |
| ÉV-38 | 3.0 | 0 | 1 | Vegetative |
| ÉV-38 | 7.5 | 0 | 3 | Vegetative |
| ÉV-38 | 2.8 | 0 | 0 | Vegetative |
| ÉV-38 | 8.5 | 0 | 1 | Vegetative |
| ÉV-38 | 6.3 | 0 | 3 | Vegetative |
| ÉV-38 | 13.1 | 0 | 0 | Vegetative |
| ÉV-38 | 2.4 | 0 | 0 | Vegetative |
| GE-01 | 21.0 | 2 | 24 | Flowering |
| GE-01 | 19.0 | 1 | 7 | Flowering |
